# Biatrial Volumetric Assessment by Non-ECG-Gated CT Pulmonary Angiography Correlated with Transthoracic Echocardiography in Patients with Normal Diastology

**DOI:** 10.3390/tomography8060230

**Published:** 2022-11-17

**Authors:** Deepa Gopalan, Jan Riley, Kai’En Leong, Senan Alsanjari, Ben Ariff, Willam Auger, Peter Lindholm

**Affiliations:** 1 Department of Physiology & Pharmacology, Karolinska Institute, 17177 Stockholm, Sweden; 2 Department of Radiology, Imperial College Healthcare, London W12 0HS, UK; 3 Department of Radiology, Monash Health, Melbourne 3168, Australia; 4 Department of Cardiology, Royal Melbourne Hospital, Melbourne 3052, Australia; 5 Department of Pulmonary Medicine, University of California, San Diego, CA 92037, USA; 6 Department of Emergency Medicine, University of California, San Diego, CA 92103, USA

**Keywords:** CT biatrial size, CT biatrial volumes, CT indexed atrial volumes, CT atrial size, CT atrial volumes, CT planimetry

## Abstract

Atrial size is a predictor of cardiovascular mortality. Non-ECG-gated computed tomography pulmonary angiography (CTPA) is a common test for cardiopulmonary evaluation but normative values for biatrial volumes are lacking. We derived normal CT biatrial volumes using manual and semiautomated segmentation with contemporaneous transthoracic echocardiography (TTE) to confirm normal diastology. Thirty-five consecutive cases in sinus rhythm with no history of cardio-vascular, renal, or pulmonary disease and normal diastolic function were selected. Planimetric CTPA measurements were compared to TTE volumes measured using area length method. TTE and CTPA derived normal LAVi and RAVi were 27 + 5 and 20 + 6 mL/m^2^, and 30 + 8 and 29 + 9 mL/m^2^, respectively. Bland–Altman analysis revealed an underestimation of biatrial volumes by TTE. TTE-CT mean biases for LAV and RAV were −5.7 + 12.0 mL and −16.2 + 14.8 mL, respectively. The CT intraclass correlation coefficients (ICC 95% CI) for LA and RA volumes were 0.99 (0.96–1.00) and 0.96 (0.76–0.99), respectively. There was excellent correlation (*p* < 0.001) between the semiautomated and manual measurements for LA (r 0.99, 95% CI 0.98–0.99) and RA (r 0.99, 95% CI 0.99–1.00). Atrial volumetric assessment on CTPA is easy and reproducible and can provide additional metric in cardiopulmonary assessment.

## 1. Introduction

There is an increasing trend to use left atrial (LA) size and function as a morphophysiologic expression to predict cardiovascular mortality in a variety of conditions such as atrial fibrillation, cardiomyopathy, ischemic heart disease, and valvular heart disease [1]. Right atrial (RA) size has also been shown to be of prognostic relevance in diverse cardiopulmonary disorders such as pulmonary embolism and pulmonary hypertension [2,3]. Cardiac magnetic resonance imaging (MRI) and echocardiography are the two non-invasive imaging techniques that are traditionally used for cardiac chamber quantification. Echocardiography is widely available and relatively inexpensive but has limited use for thoracic evaluation. MRI, with its unrivalled versatility, is the reference standard [4] and uses steady-state free precession cine imaging rather than angiographic sequences for chamber measurements as it provides high signal contrast between the ventricular blood pool and the myocardium with improved performance for semiautomated edge-detection algorithms. In spite of its numerous advantages such as operator independence, no exposure to ionising radiation, and lack of geometric assumptions to estimate heart size, MRI is not the introductory modality in the investigation of cardiopulmonary disorders and is also resource intensive and time consuming.

Non-ECG-gated computed tomography pulmonary angiography (CTPA) is a commonly performed test for assorted indications pertaining to the cardiopulmonary system including the assessment of dyspnoea and chest pain. Historically, cardiac chamber assessment on CTPA was performed subjectively but with recent technical advances, it is possible to accomplish objective evaluations. However, there is limited published data regarding normal atrial size on CTPA. There are a number of ECG-gated CT angiography [5,6,7] based publications on atrial volumetry but this literature serves to illustrate the methodological differences between the different groups. Such lack of consensus translates to difficulty in establishing normative values. In a recent systematic review by Zuin et al. [8] of the potential role of LA size derived by non-ECG-gated CT angiography in patients with acute pulmonary embolism, LA volumes were evaluated in four studies [9,10,11,12] but none had comparative TTE that could be used for corroboration of the volumes. The results were further limited by the lack of data regarding the presence of atrial fibrillation in the analysed patients.

Given the wide variation in the CT angiography methodology used for atrial measurements, both in terms of the parameters used for estimating atrial size as well as in the techniques employed for the measurements, we performed a small pilot study to examine the feasibility and reproducibility of deriving normative biatrial volumes on CTPAs using both manual contouring and semiautomated volume segmentation with comparative transthoracic echocardiography (TTE) to confirm normal diastology.

## 2. Materials and Methods

### 2.1. Patient Population

This retrospective study was approved by the Institutional Review Board and need for informed consent was waived. Internal radiology information system/picture archiving and communication system, echocardiography and electronic medical records databases were scrutinised over a 3-year period to select patients who were in sinus rhythm, had no prior history of cardiovascular, renal, or pulmonary disease, and had undergone both non-ECG-gated CTPA and transthoracic echocardiography (TTE) within a 6 month period, irrespective of the indications for the 2 tests.

**Figure 1:**
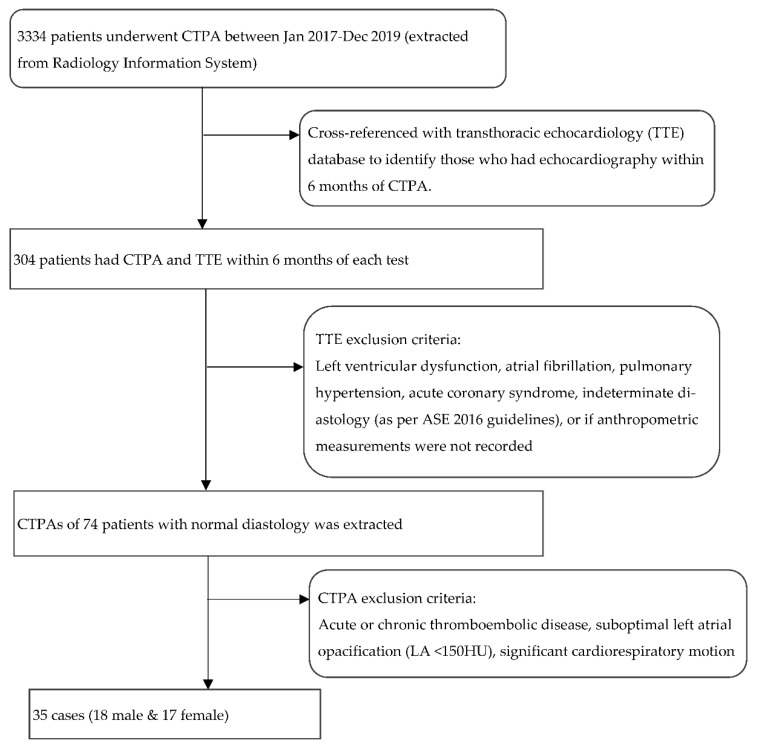
Flow chart outlining the selection process including the exclusion criteria. TTE: transthoracic echocardiography; LA: left atrium. HU: Hounsfield unit.

TTE was first analysed in accordance with current guidelines [13]. Strict exclusion criteria were then applied to identify patients with completely normal diastology (Figure 1: Flow chart of the selection process including exclusion criteria; although age was not an exclusion criterion, all patients with normal diastology in the final analysis were less than 56-years old). If the LV diastolic function was completely normal on TTE, the corresponding CTPA was then retrieved. After excluding cases with thromboembolic disease, the images were analysed for cardiorespiratory motion and contrast opacification that could adversely affect atrial size quantification. As adequate biatrial contrast enhancement is a prerequisite to both manual and semi-automated segmentation, CTPAs with low left atrial enhancement (<150 HU) were excluded. The final analysis included thirty-five cases.

### 2.2. CT Acquisition

CTPA’s were acquired on single-source 128-multislice configuration (Somatom Definition AS+; Siemens AG, Munich, Germany). Non-ECG-gated scanning was performed in craniocaudal direction from lung apices to bases at end-inspiration during a single breath-hold using the following acquisition parameters; table speed 61.4 mm/rotation, pitch 0.8 pitch, tube voltage 80–120 kVp, tube current 100 mAs, rotation time 0.5 s, and 512 × 512 acquisition matrix. A total of 100 mLs Omnipaque 350 (GE Healthcare, Chicago, IL, USA) was injected at 5 mL/s with 20-mL saline chaser. Pulmonary artery visualisation was optimised using an automated bolus-tracking technique with region of interest (ROI) placed within main pulmonary artery and trigger values of 130 Hounsfield units. Images were reconstructed at 1-mm slice thickness at 1 mm interval using SAFIRE (Sinogram Affirmed Iterative Reconstruction, strength 3) iterative reconstruction.

### 2.3. CT Biatrial Measurement

For manual measurements, CT images were analysed using Vitrea Advanced Visualization multimodal platform (Vital Images, Inc.; Minnetonka, MN, USA). CT parameters were measured in consensus by two radiologists (R1, a cardiovascular radiologist with 15 years’ experience, and R2, a radiology imaging fellow).

**Figure 2:**
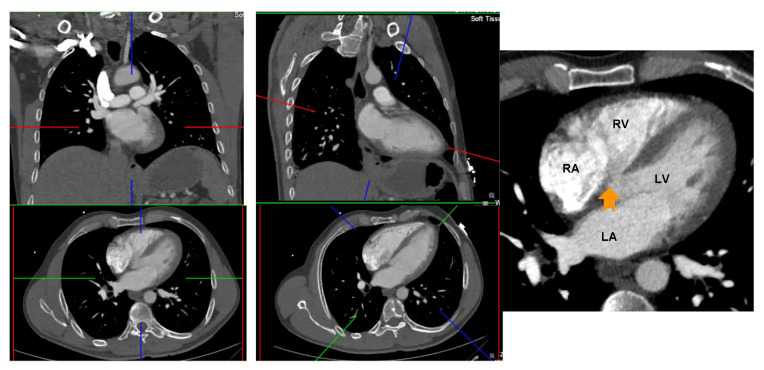
Creation of 4 and 2 chamber planes on non-ECG-gated CTPA. Left panel: Source data prior to image manipulation. Middle panel: Alignment of crosshairs with long axis reference line through LV apex and mid mitral valve and short axis reference line parallel and aligned with mitral annular plane. Right panel: Avoid creation of a 5 chamber view by adjusting the image height (arrow points to left ventricular outflow tract). RA: right atrium; LA: left atrium; RV: right ventricle; LV: left ventricle.

CT 2 and 4 chamber planes were created analogous to TTE views. Using multiplanar reformatting (MPR) with true axial stack, 2 and 4 chamber planes were created by positioning long axis reference line through LV apex and mid mitral valve, and short axis reference line parallel and aligned with mitral annular plane. Imaging slice height was adjusted cranio-caudally to remove LV outflow tract/aortic root from view, to avoid creation of TTE equivalent ‘5 chamber view’. The LV short axis plane was used for cross reference to ensure the manually created 2 and 4 chamber planes transected the appropriate and relevant myocardial segments; 2 chamber—anterior and inferior segments, 4 chamber—inferoseptum and anterolateral segment (Figure 2).

**Figure 3:**
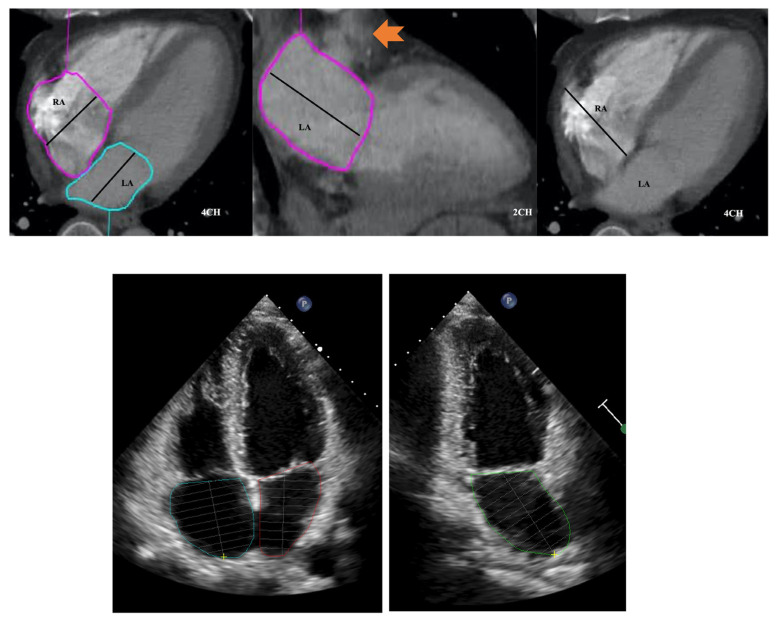
Four and two chamber planes from a non-ECG-gated CTPA (**top**) and corresponding echocardiograpy images (**bottom**). Atrial areas planimetered with exclusion of right and left atrial appendages and pulmonary veins. Arrow in middle panel indicates left atrial appendage. RA: right atrium; LA: left atrium.

**Figure 4:**
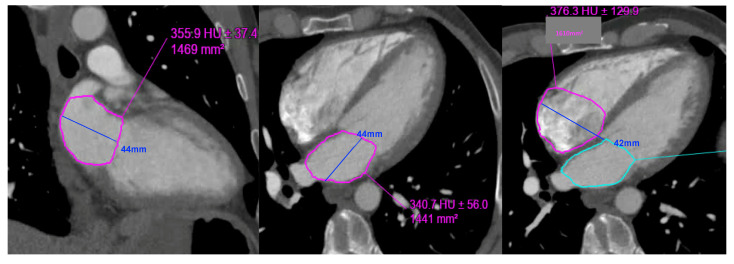
Example of left and right atrial planimetry in a 50-year-old male with body surface area of 1.9 (height 173 cm, weight 76 kg).

LA area planimetry was performed using freehand ROI tool. Pulmonary veins and LA appendage were excluded. Using TTE area length method, LA volume was estimated by: (0.85 × area 1 (2 chamber) × area 2 (4 chamber)) ÷ shortest LA long axis length [14]. Whilst limitations of area length method (relating to LA shape geometric assumptions) are acknowledged, it was selected over the Simpson modified biplane method to allow expeditious CT LA volume estimation without the need for additional software computation. Direct LA area was not measured from the straight axial stack due to divergence of LA long axis plane from the standard axial plane which would result in a degree of systematic bias and limit direct comparability with TTE. This would be further compounded by single plane 4 chamber LA measurements being smaller than 2 chamber [15]. RA volume was also measured from same CT 4 chamber plane without alteration of the horizontal imaging plane to emphasise the RA. Area planimetry was performed using freehand ROI tool with exclusion of RA appendage. Single plane RA volume was estimated by: (0.85 × (RA 4 chamber area)2) ÷ RA long axis length [14,16]. (Figure 3 and Figure 4).


Left atrial volume: (0.85 × area 1 (2 chamber) × area 2 (4 chamber)) ÷ shortest LA long axis length:0.85 × 14.7 × 14.4 ÷ 4.4 = 40.8 mL. LAVi = 21.4 mL/m^2^Right atrial volume: (0.85 × (RA 4 chamber area)2) ÷ RA long axis length:0.85 × (16.1) 2) ÷ 4.2 = 52.45 mL. RAVi: 27.6 mL/m^2^


LAVi: Left atrial volume indexed; RAVi: Right atrial volume indexed.

Semiautomated biatrial measurements were performed offline using commercially available software (CVI42 version 5.12.1, Circle Cardiovascular Imaging, Calgary, AB, Canada). The biatrial endocardial borders were manually delineated in the apical four- and two-chamber views using a point-and-click approach before the automated tracking algorithm was applied. The pulmonary veins and atrial appendages were again excluded from the analysis. Maximum and minimum volumes were calculated based on the biplane area-length method [14] and indexed to body surface area.

The time required to load the CT data into the PACS and software interface and to complete the manual and semiautomated image analyses was also recorded.

To maintain methodological comparability with CT, atrial volumes on TTE were measured using the atrial length method by a single experienced echocardiologist (KL).

### 2.4. Statistical Analysis

Continuous normal data are presented as mean (±1 standard deviation). Non-normal data are presented as median (inter-quartile range). Categorical variables are displayed as *n* (%). Continuous data was compared with the (paired) two tailed Student’s *t* test. Intraclass correlation coefficient (ICC) estimates and their 95% confidence intervals for inter-observer variability in manual CT atrial measurement were calculated. TTE and manual CT measurement of biatrial volumes was compared with Bland–Altman analysis. Manual CT and semiautomated CT measurement of biatrial volumes was also compared with Bland–Altman analysis. Correlation analysis was performed using Pearson correlation coefficient. A *p*-value of <0.05 was considered statistically significant.

## 3. Results

The baseline characteristics and TTE features demonstrating normal diastology are delineated in Table 1 and Table 2, respectively. Manual CT and TTE biatrial measurements are outlined in Table 3.

### 3.1. TTE versus CT Biatrial Volumes

**Figure 5:**
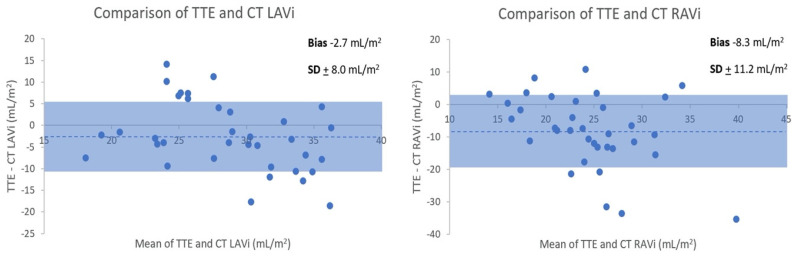
Bland–Altman comparison of normal transthoracic echocardiography (TTE) and CT (indexed) LA and RA volume measurements.

TTE and CTPA derived normal LAVi and RAVi were 27 + 5 and 20 + 6 mL/m^2^, and 30 + 8 and 29 + 9 mL/m^2^, respectively. Bland–Altman analysis (Figure 5) revealed an underestimation of biatrial volumes by TTE compared to CT. TTE-CT mean biases for (non-indexed) LAV and RAV were −5.7 + 12.0 mL and −16.2 + 14.8 mL, respectively. The intraclass correlation coefficients (ICC 95% CI) for the two readers for CT manual atrial measurements were 0.99 (0.96–1.00) and 0.96 (0.76–0.99) for LA and RA volumes, respectively.

Shading indicates ±1 standard deviation. Dotted line denotes mean bias.

Whist majority of cases had smaller LAV with TTE, some patients had larger volumes with TTE than CT. This would relate to TTE LAV being measured in end-systole at maximal atrial volume and CT being ungated. LA: left atrium. RA: LAV: left atrial volume. Right atrium. RAV: right atrial volume.

### 3.2. Manual versus Semiautomated CT Biatrial Volumes

The indexed manual and semiautomated biatrial volumes are outlined in Table 4.

**Figure 6:**
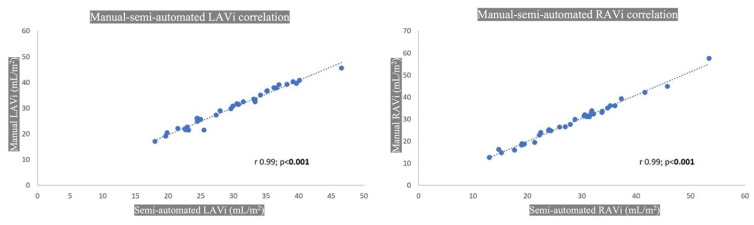
CT manual and semiautomated atrial volume correlation. LAVi: left atrial volume indexed; RAVi: right atrial volume indexed.

Excellent correlation (*p* < 0.001) was seen for LA (r 0.99, 95% CI 0.98–0.99) and RA (r 0.99, 95% CI 0.99–1.00), as displayed in Figure 6.

The mean time for biatrial analysis with the manual approach was 12 min (+/−3 min). Automated image analysis was faster with an average of 6–8 min.

## 4. Discussion

Due to the paucity of published CTPA literature on normative atrial volumes, we performed this pilot study and derived biatrial volumes on 35 patients with comparative volumes on contemporaneous TTE. Our results showed excellent correlation between CT manual and semiautomated volumetric analysis for both atria. However, compared with CT, there was an underestimation of biatrial volumes by TTE. This is not surprising as previous studies using ECG-gated CT datasets have shown similar results [17,18,19].

Quantifying LA size is difficult, in part because of the complex geometry and the variable contributions of its appendage and pulmonary veins. Furthermore, atria have an amorphous morphology and their dimensions are affected by parameters such as age and body mass index [20,21,22]. LA volume increases markedly in patients with severe renal dysfunction [23]. Taking these factors in to consideration, we carefully selected our TTE-CTPA cohort of normal diastology, ensuring normal renal function and sinus rhythm. Although we did not set an age cut-off, patients with normal diastolic function on the TTE were younger, with an upper limit of 55 years.

Axial diameter has previously been shown to be a simple and quick CT measurement of LA size with a cut-off of 4.0 cm for predicting diastolic dysfunction with a sensitivity of 68% and a specificity of 74% [24]. However, this and other studies [24,25,26] acknowledge that volumetric analysis provides a more accurate representation of LA size. Furthermore, in patients with sinus rhythm, indexed LA volume is a more robust marker of cardiovascular events than LA area or diameter [27]. This is because LA is an asymmetrical cavity and so volumetric analysis gives a more precise size estimation. Finally, LA dilatation might not be evenly distributed in all planes, and so a simple antero-posterior dimension is likely to be insensitive to LA size change [25].

Whilst there are established reference ranges for normative RV volumes on TEE, there is very limited data on measurement of RA size and in particular, RA volumes by CT. A cut-off of >35 mm, in the transverse diameter, perpendicular to the interatrial septum, measured from septal wall to lateral wall has been proposed for RA enlargement, but this is based on historic data [28]. Few studies have used ECG-gated CT datasets for RA volumetry [6,29,30] and have shown that similar to LA, the CT-derived RA volumes also tend to be overestimated when compared with MRI [29] and echocardiography [30]. The differences in the temporal resolution may account for the inter-technique variability in the measurements. A recent prospective study of 609 patients with acute PE [2] demonstrated the feasibility of performing fully automated RA volumetric analysis on non-ECG-gated CTPA. Whilst RA dilation was a frequent finding in this population, its prognostic performance was inferior compared to other risk stratification markers. Moreover, in the above study [2], there were patients with co-morbidities that affected the atrial volumes (elderly male patients with cardiovascular comorbidities had higher volumes whilst cancer patients had lower volumes) and only 25% of the cases had corresponding echocardiography which adds to the difficulty of comparing CT measurement accuracy to other techniques.

The complex RA anatomy and the suboptimal delineation of the RA wall on the CT due to streak artifacts from the intravenous contrast medium contribute to the difficulties in RA volumetric analysis. Furthermore, the RA size may be affected by the high flow rate of the administered contrast medium, as well as alterations in venous return with inspiratory breath-holds. Notwithstanding these challenges, we were able to perform both a manual and semiautomated assessment of the RA volumes with excellent correlation between the two methodologies. Similar to LA volumes, TTE underestimated the RA volumes compared to CT but with a higher magnitude of difference. This greater underestimation of RA volumes with TTE is likely due to the lack of right ventricle (RV)/ RA focused imaging as in routine clinical practice; the standard TTE four chamber imaging emphasises the left heart.

Our study has demonstrated the feasibility and reproducibility of deriving normative values on routine CTPA. A main limitation is the small sample size. We identified 304 patients with contemporaneous CTPA and TTE but a large proportion in this retrospective study group was not suitable due to diastolic dysfunction. However, it must be emphasised that the current sample size of 35 patients satisfies the central limit theorem to demonstrate Gaussian/normal distribution for assessment of CT atrial normative values. Moreover, to our knowledge, it is the only study to have corroborative TTE in all cases to ensure completely normal diastology. We also acknowledge that atrial chamber quantification is gender specific, but our work is intended as a pilot study and Gaussian distribution cannot be demonstrated if our small cohort is divided into male and female subgroups. Whilst ECG-gating may potentially improve the accuracy of the atrial volumes, we believe our approach reflects real-world clinical practice as most institutions perform CTPA without ECG-gating. Manual atrial volumetric assessment is operator dependent but our quantitative data demonstrated excellent intraclass correlation. However, whilst manual measurements without recourse to expensive and dedicated analysis software were shown to be as good as the more sophisticated semiautomated measurements, the latter are quicker and simpler to perform and hence more easily adaptable in clinical practice.

In conclusion, atrial measurements on CTPA are undergoing an evolutionary process from subjective evaluation to objective quantification. Knowledge of normal values of atrial volumes is required to differentiate between pathological conditions and normal state as well as grade the disease severity and monitor treatment response. The present work has shown that CT atrial volumetric assessment is easy and reproducible and can provide an additional metric in the CTPA assessment of cardiopulmonary diseases. Current trends using artificial intelligence algorithms are apposite for automated atrial volumetric analysis to be incorporated into routine practice. However, prospective large volume studies will be needed to validate the normative atrial volumes on CTPA.

## Figures and Tables

**Table 1:** Baseline characteristics of the thirty-five cases.

	Normal Diastology (n = 35)
**Age (years)**	45 (34–55)
**Gender**	18 M (51%), 17 F (49%)
**BMI**	27.0 (23.5–32.0)
**BSA**	1.95 ± 0.25
**CTPA-TTE time interval (days)**	44 (11–113)

BMI: body mass index; BSA: body surface area; CTPA: CT pulmonary angiography; TTE: transthoracic echocardiography.

**Table 2:** Transthoracic echocardiography (TTE) measures of normal diastology.

	Normal Diastology Cohort (n = 35)
**Simpson’s biplane LVEF (%)**	61 ± 4
**Transmitral E (cm/s)**	84 (63–96)
**Transmitral A (cm/s)**	65 (53–73)
**E/A**	1.29 ± 0.24
**Medial mitral e’ (cm/s)**	11 (9–12)
**Lateral mitral e’ (cm/s)**	14 (12–15)
**E/medial e’**	7.9 ± 2.0
**E/lateral e’**	5.9 ± 1.4
**TR Vmax (m/s) (n = 15)**	2.3 ± 0.2

LVEF: left ventricular ejection fraction. TR: tricuspid regurgitation.

**Table 3:** Manual CT and transthoracic echocardiography (TTE) atrial measurements.

	CT Atrial Measurements (Manual)	TTE Atrial Measurements
**4Ch LA area (cm^2^)**	18.1 ± 4.1	18.3 ± 3.4
**2Ch LA area (cm^2^)**	17.0 ± 4.2	16.7 ± 3.8
**LAVi (mL/m^2^)**	30 ± 8	27 ± 5
**4Ch RA area (cm^2^)**	17.0 ± 4.1	15.1 ± 3.2
**RAVi (mL/m^2^)**	29 ± 9	20 ± 6
**4Ch RA:LA area ratio**	0.91 (0.82–1.06)	0.84 ± 0.14
**RA:LA volume ratio**	0.88 (0.81–1.09)	0.76 ± 0.23

LAVi: left atrial volume index; RAVi: right atrial volume index.

**Table 4:** Manual automated CT atrial measurements.

	Manual Measurement (n = 35)	Semiautomated Measurement (n = 35)	*p*-Value
**LAVi (mL/m^2^)**	30 ± 8	30 ± 7	NS
**RAVi (mL/m^2^)**	29 ± 9	28 ± 9	NS

LAVi: left atrial volume index; RAVi: right atrial volume index.

## Data Availability

The data presented in this study are available on request from the corresponding author. The data are not publicly available as the study was not registered on a public domain.
